# Expert Group Recommendations on the Effective Use of Bolus Insulin in the Management of Type 2 Diabetes Mellitus

**DOI:** 10.3390/medsci9020038

**Published:** 2021-05-28

**Authors:** Rajeev Chawla, Jagat Jyoti Mukherjee, Manoj Chawla, Alok Kanungo, Meenakshi Sundaram Shunmugavelu, Ashok Kumar Das

**Affiliations:** 1 Department of Diabetology, North Delhi Diabetes Centre, 180, Jai Apartments, Sec 9, Rohini 110085, India; rajeevaastikchawla@yahoo.com; 2 Division of Endocrinology, Department of Medicine, Apollo Gleneagles Hospitals, 58, Canal Circular Road, Kolkata 700054, India; 3 Lina Diabetes Care and Mumbai Diabetes Research Centre, 704, Cosmos Plaza, Opp. Indian Oil Nagar, J.P. Road, Andheri (W), Mumbai 400053, India; drmanojchawla@yahoo.com; 4 Department of Diabetology, Kanungo Institute of Diabetes Specialities Pvt. Ltd., 1120, Dumduma, Bhubaneswar 751019, India; kanungokids@gmail.com; 5 Department of Diabetology, Trichy Diabetes Speciality Centre (P) Ltd. B-37, Sasthri Road, VII Cross East, Thillai Nagar, Trichy 620018, India; msv_diab@yahoo.com; 6 Department of Internal Medicine, Pondicherry Institute of Medical Sciences, Kalathumettupathai, Ganapathichettikulam Village, No 20, Kalapet, Puducherry 6050146, India; ashokdas82@gmail.com

**Keywords:** bolus insulin, type 2 diabetes mellitus, expert group recommendations

## Abstract

Evidence suggests a major contribution of postprandial glucose (PPG) excursions to the increased risk of micro- and macro-vascular complications in individuals with type 2 diabetes mellitus (T2DM). Administration of bolus insulin remains a very effective therapeutic option for PPG control. The aim of this expert group recommendation document was to provide practical and easy-to-execute guidelines for physicians on the appropriate use of bolus insulin in the management of T2DM. A panel of key opinion leaders from India reviewed and discussed the available clinical evidence and guideline recommendations on the following topics: (1) optimum control of PPG; (2) choice of bolus insulin; and (3) special situations and practical considerations. The expert panel critically analyzed the current literature and clinical practice guidelines and factored their rich clinical experience to develop a set of nine expert group recommendations for the effective use of bolus insulin. These recommendations will not only result in a more evidence-based application of bolus insulin in the clinical setting but also trigger further research and provide a valuable base for the development of future guidelines on the use of bolus insulin in the management of individuals with T2DM.

## 1. Introduction

In the year 2017, India and China recorded the highest number of individuals with diabetes within the age group of 20–79 years (72.9 million and 114.4 million, respectively) [1]. Type 2 diabetes mellitus (T2DM) is the most common form of diabetes, and is a complex, progressive metabolic condition marked by anomalous insulin production and utilization [1,2]. Indian patients with T2DM have distinct characteristics, leading to the moniker “Asian Indian phenotype”. When compared to the Western population, they have a younger age of onset; relatively high body-fat percentage, waist-to-hip ratio, and insulin resistance; a rapid decrease in β-cell function; and a high predisposition to cardiovascular disease (CVD) [3,4,5,6]. Furthermore, risk factors such as sedentary lifestyle, consumption of high-sugar diets, and increased total dietary carbohydrate intake contribute to an increased risk of T2DM in Indians [7,8]. Despite the high prevalence estimates and prevailing risk factors, the Indian Council of Medical Research–INdia DIABetes (ICMR-INDIAB) study phase 1 report showed that only 30.0–34.7% of urban diabetes population and 17–40% of the rural diabetes population have good glycemic control (glycosylated hemoglobin (HbA1c) < 7%) [9].

The deterioration of glucose homeostasis in T2DM follows a three-step process, wherein a gradual loss in daytime postprandial glucose (PPG) control precedes the deterioration in fasting plasma glucose (FPG) control [10]. Optimum management of a person with T2DM calls for a thorough understanding of the relation between FPG, PPG, and HbA1c [11]. A target HbA1c value of <7% has long been the driving factor for efficient management of T2DM; however, evidence over time has pointed towards the role of PPG in T2DM, as it serves to be the main contributor of hyperglycemia in fairly-controlled patients with T2DM [2,12,13]. The key determinants of overall PPG control are pre-prandial glycemic levels; insulin sensitivity; meal size and content; gastric emptying; incretin hormones; and insulin and glucagon secretion [11,14,15]. Along with basal hyperglycemia, PPG excursions also lead to an increase in the overall glucose exposure [16]. PPG has been shown to be an important predictor of micro- and macrovascular complications and mortality in individuals with T2DM [16,17,18]. Epidemiological studies around the globe have further provided qualitative evidence on the correlation of increased PPG and CVD [19,20,21,22,23].

In the light of the above facts, various global guidelines advocate monitoring of PPG, and attaining specific PPG values for achieving optimum glycemic control [2,16,24,25]. Therefore, clinicians should not only aim at controlling FPG but also emphasize on controlling the PPG when deciding treatment strategies for effective management of individuals with T2DM.

Prandial or bolus insulin is one of the options for control of PPG excursions in individuals with T2DM [2,26,27]. A summary of the various prandial/bolus insulin preparations used for PPG control, along with their brief pharmacodynamic (PD) profiles, is presented in Table 1 [27,28].

Since 2009, several expert group recommendations and evidence-based guidelines on the various aspects of use of insulin in individuals with diabetes mellitus have been published in India. These recommendations guide the treating clinicians on the choice, dose, and intensification of insulin. However, most such recommendations and guidelines have either focused on premixed [29,30,31] or basal insulin regimens [32,33]. Currently, there are no comprehensive guidelines/expert group recommendations on the use of bolus insulin in the management of T2DM in India. Hence, we set out to develop a consensus document on the choice, dosing, and titration of bolus insulin in the management of T2DM.

## 2. Materials and Methods

Evidence for the expert group recommendations was assimilated from the published literature in PubMed through a comprehensive search of clinical trials (*n* = 41), observational studies (*n* = 34), meta-analyses (*n* = 6), review articles (*n* = 30), global and Indian guidelines related to management of T2DM (*n* = 24) on postprandial glucose, and the use of bolus insulin. These lines of evidence were thoroughly reviewed, analyzed, and presented by key opinion leaders at the 12th National Insulin Summit, held in Hyderabad, India, on the 8th of September 2018. The expert panel comprised of endocrinologists, clinicians, and key opinion leaders from various regions of India. The committee reviewed and exchanged views on each topic relating to T2DM management with bolus insulin, deriving several recommendations. For topics having little or no evidence, the experts relied on clinical experience and unanimity to formulate the recommendations. The recommendations were also tailored keeping in mind the prevalent therapeutic needs and practices.

The final expert group recommendations on the use of bolus insulin for the treatment of T2DM were proposed and recorded in an unbiased manner. The document was drafted and disseminated among the experts for their final feedback. 

### The Grading System

The present expert group recommendation document is built in conformity with the American Diabetes Association (ADA) protocol for grading of scientific evidence [2,34]. The recommendations provided in this document have been assigned ratings of grades A, B, or C depending on the quality of supporting evidence, as described by ADA. For evidence Grade A, in addition to the ADA-based description, the following were also regarded as grade A recommendations: Recommendations strongly supported by evidence-based, national and international clinical practice guidelines, including Research Society for the Study of Diabetes in India (RSSDI), ADA, International Diabetes Federation (IDF), and American Association of Clinical Endocrinologists (AACE). This categorization of recommendations are supported strongly by evidence-based guidelines as grade A based on the 6S model of per-appraised evidence. This model includes clinical practice guidelines based on comprehensive or systematic searches and appraisal of literature, at one of the highest layers of evidence, even above meta-analyses and original studies, and recommends the use of strong recommendations supported by high-quality evidence from these guidelines [35,36]. The grade E is provided for recommendations, which do not have adequate supporting evidence from clinical trials and are based on expert opinion. The detailed evidence-grading system for the proposed recommendations is shown in Table 2. The ethical committee approval is not required for this project as it is not dealing with any patient data. 

## 3. Results

### 3.1. Optimum Control of Postprandial Hyperglycemia

#### 3.1.1. Scientific Evidence: Clinical Implications of PPG

Impact of PPG on Overall Glycemic control: The relative contribution of FPG and PPG to overall glycemic control has been a topic of debate. A study conducted among 290 individuals with T2DM in Europe revealed that the contribution of PPG excursions is predominant when T2DM is fairly controlled, i.e., when the HbA1c quartiles are low, whereas the contribution of FPG is progressively increased with worsening diabetes control, i.e., when the HbA1c quartiles are high [13]. However, the scenario is different in Asia. A study conducted using continuous glucose-monitoring (CGM) among non-insulin-taking Asian patients with T2DM demonstrated that PPG not only contributed substantially in lower HbA1c quartiles but also showed a similar contribution to FPG at higher HbA1c quartiles. This study is more relevant to the Indian population because:

- Treatment similarity: About one-third of patients were treated with metformin + sulfonylurea and another one-third of patients were treated with metformin + acarbose. This treatment regimen is similar to the regimen taken by Indian patients.
- Body mass index (BMI) similarity: Majority of patients in this study had a BMI < 27 kg/m^2^. This picture is similar to what is seen among Indian patients and different from the Caucasian population, who have a relatively higher BMI.
- More accurate measurement of the contribution of PPG and FPG to HbA1c: This study used CGM, following a real-life meal as opposed to 2-h plasma glucose measurement after a standard meal or oral glucose load as used in other studies. It is also worthwhile to note that unlike CGM, 2-h PPG measurement might miss the peak glucose excursion, which might result in underestimation of PPG contribution to overall HbA1c. Hence, it is important to note that controlling PPG is as important as controlling FPG in Asian patients with a mean HbA1c of up to 12.7% [37].

The importance of PPG control in achieving HbA1c targets has also been highlighted in several other studies. A meta-analysis by Ketema et al. to understand the correlation of PPG and FPG with HbA1c found PPG to be a better predictor of overall glycemic control compared to FPG in the absence of HbA1c. About 63.5% of the studies included in the analysis showed a significantly stronger correlation of PPG with HbA1c. Furthermore, PPG demonstrated better specificity, sensitivity, and positive predictive value than FPG [38]. In a prospective, interventional study, control of FPG was found to be insufficient for achieving HbA1c goals <7%. While 94% of patients achieving the PPG target <140 mg/dL achieved the HbA1c goal <7%, only 64% of patients achieving the FPG target <100 mg/dL achieved the set HbA1c target. The decrease in PPG values accounted for approximately twice as much for the decline in HbA1c as did decreases in FPG. The study revealed that when HbA1c was <6.2%, PPG accounted for close to 80% of HbA1c values and approximately 40%, when the HbA1c level was >9% [39]. Therefore, guidelines also recommend that controlling PPG is important to achieve HbA1c goals [40].

Impact of PPG on Long-Term Diabetes Complications: Apart from contributing to overall glycemic control, control of PPG is important to prevent micro- and macrovascular complications. In a study conducted in 726 South Indian T2DM patients with a diabetes duration of more than 25 years, retinopathy, nephropathy, and neuropathy were detected in 52%, 12.7%, and 69.8% of the patients, respectively. Multivariate regression analysis revealed that postprandial hyperglycemia was associated with 2.4 times higher risk of nephropathy and 79% increased risk of neuropathy [18]. Studies have revealed a consistent and significant correlation between postprandial hyperglycemia and the risk of CVD events and mortality [17,19,20,21,22,23,41]. Moreover, the risk of CVD and associated mortality is higher in Indians and Asian Indians compared to other populations [42,43,44,45]. Some of the pathophysiological mechanisms explaining the correlation between increased PPG and CVD risk include production of free radicals, increase in oxidative stress, endothelial function disorder, low-grade inflammation, blood coagulation, reduction in fibrinolysis and early-phase insulin secretion, and increased insulin resistance [21,23].

Criticality of PPG in the Indian Scenario: In a study conducted on 52,419 patients with T2DM, PPG excursions were found to be the highest among Indians (277 mg/dL) as compared to the rest of the world (Canada, Russia: 212 mg/dL; Italy: 171 mg/dL; Poland: 207 mg/dL; Greece: 248 mg/dL) [46]. A plausible explanation for this finding may be the consumption of high-calorie/high-fat and high sugar diets in India [7]. The percentage of energy derived from carbohydrate in both rural and urban India is over 70% [47]. In this context, it is pertinent to mention that the postprandial glycemic load has been found to be proportionate to carbohydrate intake or glycemic index of diets [8,48].

#### 3.1.2. Scientific Evidence: Importance of Controlling PPG in People with T2DM as Highlighted in Various Guidelines/Recommendations

The IDF guideline for the management of post-meal glucose in diabetes clearly states that post-meal hyperglycemia leads to significant short-term and long-term complications and requires utmost attention in patients with T2DM [49]. The recently published American Diabetes Association (ADA) standard of care on diabetes acknowledges that postprandial hyperglycemia contributes to elevated HbA1c values, with its relative contribution being greater at HbA1c values closer to 7%. Further, the guideline states that high post-challenge glucose values are associated with an increased risk of CVD, independent of FPG values. The guideline adds that in individuals with diabetes, endothelial dysfunction, one of the surrogate measures of vascular pathology is negatively affected by post-meal hyperglycemia [2].

Recent clinical practice recommendations by the RSSDI for the management of T2DM echo similar thoughts on the impact of PPG. The RSSDI recommendations urge for the implementation of treatment strategies to lower PPG in individuals with post-meal hyperglycemia. Additionally, the guideline recommends the monitoring of the efficacy of treatment regimen as frequently as needed in order to guide the therapy towards achieving PPG targets [25].

#### 3.1.3. Expert Group Recommendations 01

➲ Postprandial hyperglycemia is a strong risk factor for increased risk of microvascular and macrovascular complications in individuals with T2DM. Grade A
➲ Postprandial hyperglycemia is of greater concern in the majority of Indians with T2DM because of increased consumption of carbohydrate-rich food. Grade E
➲ Treatment strategies for the management of T2DM should aim to effectively control not only fasting but also postprandial hyperglycemia. Grade A

### 3.2. Right Time to Control PPG

#### 3.2.1. Scientific Evidence

Studies indicate that pancreatic insulin release may be initially stimulated by cephalic phase metabolic responses, which are triggered by the sight, smell, and taste of the meal [50]. Later, insulin secretion by the pancreatic β-cells occurs in a biphasic manner. The first/early phase of insulin secretion is critical in maintaining the PPG homeostasis, and can prevent chronic postprandial hyperglycemia [51,52,53]. The second phase of insulin secretion is gradual and is dependent on the degree and duration of glucose stimulation [51,52].

The first phase insulin secretion inhibits hepatic glucose production early in the absorptive state, whereas the second phase insulin secretion reduces the postprandial excursions through the promotion of glucose uptake by peripheral tissues. Loss of first-phase insulin secretion is an independent predictor of onset of T2DM. As the first phase of insulin secretion is lost in T2DM, second phase insulin secretion is further impaired, causing postprandial hyperglycemia, which serves as an early marker of T2DM progression [51,54]. Considering the fact that T2DM begins with meal-related hyperglycemia in many patients, it becomes apparent to target the normalization of PPG levels as one of the goals in the early stages of the disorder [53].

In many patients with T2DM in need of insulin for achieving a glycemic goal, insulin therapy is initiated with intermediate or long-acting insulin, and the starting insulin dose is individualized based on patient profile and duration of disease [55]. However, increasing the dose of basal insulin alone shall not help achieve glycemic target because it primarily targets FPG and PPG remains untargeted for. Furthermore, an increase in the basal insulin dose will also put the patient at risk of hypoglycemia [56]. To target PPG, addition of a bolus insulin is one of the options [57]. 

A 26-week randomized study was conducted among insulin-naive patients with T2DMon ≥ 2 oral antidiabetic treatments to compare the effectiveness and safety of basal insulin (insulin glargine) (*n* = 149) versus insulin degludec aspart co-formulation (IDegAsp), which is a combination of ultra-long-acting insulin (insulin degludec) and a rapid-acting insulin analogue (RAIA) (insulin aspart) (*n* = 147). A significantly larger proportion of patients on IDegAsp achieved the HbA1ctarget of <7% (ADA goal) without confirmed hypoglycemia compared to those on basal insulin alone (43% vs. 25%, respectively, *p* < 0.01) [55]. Another 18-week, randomized, open-label study was conducted to understand the effect of basal bolus (BB) vs. basal-only insulin regimen on glycemic control in 236 patients with inadequately controlled T2DM on basal insulin and oral antidiabetic drugs (OADs). Patients were randomized to either continuing basal insulin regimen or receiving basal insulin along with mealtime fast-acting insulin aspart (BB), both with metformin. The BB regimen was found to be superior to basal-only regimen as assessed by reduction in HbA1c; the HbA1c decreased from 7.9% to 6.8% in the BB group as opposed to from 7.9% to 7.7% in the basal-only group (estimated treatment difference (ETD): −0.94%, 95% confidence interval (CI): −1.17, −0.72; *p* < 0.0001). A higher proportion of patients achieved the glycemic target (HbA1c < 7%) without severe hypoglycemia in the BB arm compared to basal alone arm (59% vs. 18%, respectively, OR: 9.19, 95% CI: 4.64; 18.20). The mean reduction in overall 2-h PPG significantly favored the BB regimen over the basal-only regimen (reduction from 180 mg/dL at baseline to 130 mg/dL at end of trial in the BB group versus from 186 mg/dL to 173 mg/dL in the basal-only group; *p* < 0.0001) [58].

It is therefore important that efforts to control postprandial hyperglycemia be initiated early in patients with T2DM and must be continued through out to achieve optimal glycemic targets.

#### 3.2.2. Expert Group Recommendations 02

➲ T2DM begins with postprandial hyperglycemia in many individuals and should be routinely monitored. Grade E
➲ Unlike Caucasians, PPG contributes significantly to the HbA1c value throughout the HbA1c spectrum ranging from 5.7% to 12.7% in Asian patients with T2DM. Grade B
➲ Early achievement of PPG targets is an important consideration for early attainment of HbA1c goals. Grade A
➲ Early interventions to minimize PPG excursions might be beneficial in reducing the risk of cardiovascular complications. Grade A

### 3.3. Time to Check and Target for PPG 

#### 3.3.1. Scientific Evidence

Compared to FPG, elevated 2-h PPG value is a stronger and independent indicator for future cardiovascular events in individuals with T2DM [20]. A 6-year, randomized study revealed a strong correlation between high 2-h PPG value and increased risk of nephropathy and retinopathy; it also highlighted the glycemic thresholds for preventing the onset and the progression of diabetes-related microangiopathy. The thresholds for HbA1c and 2-h PPG were <6.5%, and <180 mg/dL, respectively. Furthermore, 2-h PPG value was found to be independently related to mortality [59]. In an epidemiological study, participants with increased 2-h PPG had an increased risk of mortality (hazard ratio (HR): 1.8 in males and 2.6 in females) [60].

Recent studies have shown that elevated 1-h PPG is also a strong predictor of progression to pre-diabetes and diabetes, and poor metabolic profile in individuals with normal glucose tolerance (NGT) [61,62,63,64,65,66]. In a retrospective study conducted in India, FPG, 1-h PPG, and 2-h PPG obtained from an oral glucose tolerance test (OGTT) performed on 1356 individuals with NGT were analyzed separately using receiver operating characteristic (ROC) curves to predict prediabetes and diabetes. It was seen that 1-h PPG was a better predictor of diabetes than HbA1c as well as HbA1c and FPG used in combination [65]. In a 30-year follow-up of the Glucose Intolerance, Obesity, and Hypertension study conducted in Israel, the 1-h PPG value >155 mg/dL predicted mortality (28% increased risk), even when the 2-h PPG was <140 mg/dL [22]. The detrimental effect of elevated 1-h PPG can be attributed to various mechanisms, such as increased arterial stiffness and left ventricular (LV) mass; LV diastolic dysfunction; and high triglyceride to high-density lipoprotein ratio and apolipoprotein B levels [61,62,63,64,65,66,67].

Three PPG values have been recommended as the target for optimal glycemic control. The three values include <180 mg/dL (as recommended by ADA 2021 standard of care) [2], <160 mg/dL (as recommended by IDF 2014) [16], and <140 mg/dL (as recommended by AACE 2020) [68].

The rationale for a PPG target of <180 mg/dL stems from the Diabetes Control and Complications Trial (DCCT). In this study, intensive therapy of diabetes, targeted at a PPG <180 mg/dL, was found to delay the onset and progression of microvascular complications such as retinopathy (by 54% and 76% in individuals with and without mild retinopathy at baseline, respectively), neuropathy (by 60%), and microalbuminuria (by 39%) [69]. The A1c-Derived Average Glucose (ADAG) study explored the average glucose values for attaining specific HbA1c values. It was found that a PPG of 171–175 mg/dL corresponds to an HbA1c of 7.0–7.5% [70]. Hence, to achieve an HbA1c target of <7%, ADA recommends PPG of <180 mg/dL.

The rationale for a PPG target of <160 mg/dL stems from the fact that although the target for PPG in DCCT was <180 mg/dL, the mean glucose value in the intensive therapy group that achieved an HbA1c <7% was 155 mg/dL [69]. Moreover, the ADAG study determined the average glucose values for attaining specific HbA1c values and found that a PPG of 163–177 mg/dL corresponded to an HbA1c value of 6.5–6.99% [70].

The rationale for a PPG target of <140 mg/dL is based on the fact that this is the cutoff value for defining impaired glucose tolerance. Based on this fact, AACE has opted for the target PPG value of 140 mg/dL [71]. It is also worthwhile to note that, in the ADAG study, the PPG value of 141–153 mg/dL corresponded to an HbA1c value of 5.5–6.49% in patients with T2DM [70]. To achieve an HbA1c target of <6.5%, the PPG target should be less than 140 mg/dL. Studies conducted in individuals without diabetes and IGT have also revealed an increased risk of CVD at PPG levels >140 mg/dL [72,73,74].

#### 3.3.2. Time to Check and Target PPG in Various Guidelines/Recommendations

Recommendations by various guidelines on the time of monitoring and target value to be achieved for PPG are shown in Table 3 [2,16,24,25,68,75,76,77,78,79]. Several guidelines also specify regarding the need for individualization of PPG goals based on the duration of diabetes, age, life expectancy, co-morbid conditions, and risk of hypoglycemia [2,16,24,25,76,77].

#### 3.3.3. Expert Group Recommendation 03

➲ PPG targets should be individualized and based on the duration of diabetes, age, life expectancy, comorbid conditions, and risk of hypoglycemia (RSSDI ABCDEFGH approach). Grade A
➲ Emerging evidence suggests that 1-h PPG can also be a target in the management of T2DM. Grade B
➲ Monitoring of PPG at 1-h or 2-h can help optimize treatment options for PPG control. Grade C
➲ In a non-pregnant adult individual, a 1-h or 2-h post-meal blood glucose value <160 mg/dL is recommended. Grade A

## 4. Choice of Bolus Insulin in Routine Clinical Practice

### 4.1. When to Choose Bolus Insulin?

#### 4.1.1. Scientific Evidence

Initiation of bolus insulin therapy in patients with T2DM should be considered when glycemic targets are not met by a combination of multiple oral hypoglycemic agents (OHA) and basal insulin regimen as depicted in the following situations:

(1) HbA1c is not at goal with 0.5 U/kg/day of basal insulin;
(2) HbA1c is elevated despite normal FPG;
(3) FPG is within target range, but PPG is beyond target;
(4) Further increase in the dose of basal insulin results in hypoglycemia [40,79].

The various options available to treat a person with T2DM who is not at target glycemic goal despite multiple OHA and basal insulin therapy are listed in Table 4, together with their physiological activity and cost comparisons.

#### 4.1.2. Choice of Insulin to Target PPG in Various Guidelines/Recommendations

The AACE recommends using either bolus or premixed insulin in combination with OHAs or glucagon-like peptide-1 receptor agonist (GLP-1RA) after the failure of basal insulin [68]. The ADA guidelines recommend initiation of bolus insulin therapy as one of the treatment options, when glycemic targets are not achieved with basal insulin therapy and OHA [2]. The other options advocated by the ADA after the failure of basal insulin include the use of GLP-1RA or premixed insulins [2]. The Canadian guideline also recommends bolus insulin when glycemic targets are not achieved on basal insulin and OHA. Furthermore, the guideline suggests preference for rapid-acting bolus insulin analogues over short-acting regular human insulin (RHI) [80].

The RSDDI guideline recommends addition of bolus insulin with the largest meal of the day following inability to maintain glycemic targets on basal insulin and OHAs. The other options recommended by RSSDI include the addition of premixed insulin therapy, co-formulation insulins, or GLP-1RA [25].

#### 4.1.3. Expert Group Recommendations 04

To achieve target PPG value, consider adding a bolus insulin before meals in:

➲ Well-motivated individuals with T2DM who are not at target PPG value on maximal oral hypoglycemic agents and basal insulin, who can self-monitor blood glucose and accordingly titrate the insulin dose. Grade A
➲ Individuals with T2DM who require separate adjustments in basal and bolus insulin doses to achieve optimal glycemic control, which is not possible with fixed dose pre-mixed insulins. Grade E
➲ Individuals with T2DM with considerable variations in day-to-day meal timings and composition. Grade E

### 4.2. Which Bolus Insulin to Consider?

#### 4.2.1. Scientific Evidence

Rapid-Acting Insulin Analogues Versus Regular Human Insulin: Choosing the right bolus insulin for the right patient is very important to achieve glycemic targets. Randomized controlled trials (RCTs) exploring the pharmacokinetic (PK) and PD properties of bolus insulins in patients with type 1 diabetes mellitus (T1DM) have demonstrated that compared to RHI, the RAIAs, aspart, lispro, and glulisine demonstrate an earlier onset of action, higher peak, shorter duration of action, and a greater glucose-lowering effect [81,82,83]. RAIA-based regimens have been found to significantly lower the:

1. 1-h and/or 2-h post-meal glucose excursions compared to RHI-based regimens in individuals with T1DM [84,85,86,87]. They have also been found to provide better HbA1c control and have relatively less risk of hypoglycemia compared to RHI-based regimens in patients with T1DM [84,88]. In a prospective randomized controlled trial, the percentage of patients with major hypoglycemia in the insulin aspart group reduced from 11% to 8% in the first and last 3 months of the treatment, while the proportion was unaffected (11%) in the human insulin group [82]. Comparison of basal-bolus regimens with bolus RAIAs versus regimens with bolus RHI in patients with T1DM has also revealed better post-meal glycemic control and HbA1c reduction [89], and fewer hypoglycemic episodes [90] with the RAIA-based BB regimens [89].
2. Similar findings have been reported in patients with T2DM. Better post-meal glucose control [91,92], and reductions in HbA1c [92,93] have been noted with RAIA versus RHI-based bolus treatment in T2DM patients. Fewer nocturnal hypoglycemic episodes, and good patient satisfaction have also been reported in T2DM patients on bolus RAIAs [91,93].
3. A double-blind, cross-over, euglycemic clamp study was conducted among 24 patients with T1DM to compare the PK-PD characteristics of insulin lispro with that of insulin aspart administered subcutaneously; both insulin lispro and insulin aspart were equivalent (80–125%) in terms of all the PK-PD parameters [94]. In the euglycemic clamp studies conducted to compare subcutaneous insulin lispro and insulin glulisine in T1DM patients, it was seen that both RAIAs showed similar PK-PD profiles; the action-time profiles of both RAIAs were superimposable [95,96].
4. A meta-analysis by Heller et al. of randomized studies in patients with T1DM and T2DM compared the efficacy of a BB regimen with RHI versus insulin aspart as bolus insulins; neutral protamine Hagedorn (NPH) was the basal insulin. The BB regimen with aspart as the bolus insulin showed minimal, but statistically significant improvement in overall glycemic control compared to that seen in the regimen with RHI as the bolus insulin. Furthermore, the insulin aspart-based BB regimen led to a reduction in the risk of nocturnal hypoglycemic episodes by 25% compared to RHI-based BB regimen [86]. In addition to a good safety profile, long-term use of insulin aspart has been found to offer a significant reduction (43%) in the rate of cumulative cardiovascular events when compared to RHI (6.4% vs. 11.1%; *p* < 0.02) in patients with T2DM [97].

Ultrafast-Acting Insulin Analogues Versus Rapid-Acting Insulin Analogues: Although RAIAs have a quicker onset of action compared to RHI, there is still a physiological gap that persists compared to the action profile of endogenous insulin secretion in response to a meal. Thus, ultrafast-acting insulins are expected to:

1. Bridge this physiological gap of RAIAs and mimic as closely as possible the action profile of endogenous insulin secretion
2. Provide better mealtime and post-meal flexibility
3. Provide a better pump profile [98].

The fast-acting insulin aspart (faster aspart) is a product of incremental research, which is obtained by addition of two excipients to the original insulin aspart formulation. These two excipients are Niacinamide (Vitamin B3—an absorption modifier) and L-arginine (a naturally occurring amino acid added for stability). Niacinamide is a hydrotrope. In higher concentration, it shifts the balance of insulin aspart from hexameric to monomeric form, which affects oligomerization. It is also known to cause transient vasorelaxation, thereby affecting the transport of insulin monomer. Hence, niacinamide makes the rapid-acting aspart insulin into an ultra-fast acting insulin [99]. In a meta-analysis of six PK and three PD studies, faster aspart has been found to offer a twice faster onset of appearance (4.9 vs. 9 min), two-fold higher insulin exposure in first 30 min and 74% greater early glucose-lowering effect in the first 30 min when compared with insulin aspart [100]. In a phase 3 clinical trial in T1DM patients, faster aspart showed a significantly greater 1-h PPG reduction compared to insulin aspart (ETD: −21.2 mg/dL (*p* < 0.0001)) [101]. A similar significantly better 1-h PPG control was also seen in patients with T2DM (ETD: −10.6 mg/dL (*p* = 0.0198)) [58]. The 2-h PPG reduction was significantly greater in T1DM in faster aspart arm compared to insulin aspart (ETD: −12.0 mg/dL (*p* = 0.0375)) [101]. A similar trend favoring faster aspart compared to insulin aspart for better 2-h PPG control (ETD: −6.6 mg/dL) was noted in individuals with T2DM but, the difference was not significant (*p* = 0.106). Faster aspart offered a significantly greater HbA1c reduction compared to insulin aspart in 26-week (ETD: −0.15%, 95% CI: −0.23; −0.07) and 52-week (ETD: −0.10%, 95% CI: −0.19; −0.00) studies in T1DM patients [101,102]. Further to this, faster aspart showed a 74% greater early glucose-lowering effect in euglycemic clamp studies and a significantly better HbA1c reduction of 0.15% compared to insulin aspart [100,101]. In the clinical trial that was conducted among patients with type 2 diabetes, faster aspart was non-inferior to insulin aspart in terms of HbA1c reduction (ETD: −0.02%, 95% CI: −0.15; 0.10). This could be due to the heterogeneity observed in patients with T2DM in terms of residual beta-cell function and insulin resistance, which are not seen in T1DM patients [58]. No difference in the overall rate of severe and blood glucose-confirmed hypoglycemia has been noted between the two insulins in both T1DM and T2DM patients [58,101,102]. In a pooled analysis of two large T1DM trials, faster aspart showed a 16% significantly lower risk of nocturnal hypoglycemia compared with insulin aspart [103]. Efficacy and safety of fast-acting insulin aspart were compared with insulin aspart in children and adolescents with T1DM. Mealtime faster aspart was superior and post-meal faster aspart was non-inferior in HbA1c reduction when compared to insulin aspart. The control of 1-h PPG increment was also significantly better with mealtime faster aspart when compared to insulin aspart [104]. In the elderly patients with T1D, fast acting insulin aspart showed an earlier onset of exposure, and a higher early insulin exposure whilst maintaining a similar total exposure and maximum concentration compared to insulin aspart. In the three controlled clinical studies, 192 of 1219 (16%) fast acting insulin aspart treated patients with type 1 or type 2 diabetes were ≥65 years of age, and 24 of 1219 (2%) were ≥75 years of age. No overall differences in safety or effectiveness were observed between elderly and younger patients [105]. Owing to the quicker onset of action and left shift of the PK-PD curve, it is clinically important to note that hypoglycemia may occur earlier in relation to a meal with faster aspart compared to insulin aspart [58,101]. Faster aspart is currently the fastest acting subcutaneous bolus insulin in India.

#### 4.2.2. Role of RAIAs in the Management of People with T2DM in Various Guidelines/Recommendations

The AACE 2020 guidelines prefer RAIAs over short-acting RHIs for bolus insulin therapy [68]. The ADA, CDA, and RSSDI guidelines also prefer RAIAs over RHI for better glycemic control [2,25,80]. RSSDI further adds that RAIAs are useful when postprandial hyperglycemia is of concern, and the preferred RAIA can be injected just before or along with the meal [25]. The highest surge in PPG with common Indian foods occurred 30–60 min after meal and mealtime insulins that can more closely match the glycemic and insulin response are of good clinical value [106].

#### 4.2.3. Expert Group Recommendations 05

➲ When the cost of therapy is not a consideration, RAIAs (aspart, lispro, and glulisine) are preferred over RHIs because of a lesser risk of hypoglycemia and greater mealtime flexibility. Grade A
➲ Ultrafast-acting insulins more closely mimic physiological mealtime insulin secretion compared to RAIAs. Grade A
➲ The ultrafast acting insulins might be better suited for the Indian scenario with high PPG excursions. Grade C
➲ Fast-acting insulin aspart, an ultrafast-acting insulin, may be preferred over RAIAs in patients with T2DM for better control of 1-h postprandial hyperglycemia with similar control at 2–4 h. Grade A

### 4.3. Dosing and Titration: Single Dose, Subcutaneous Administration

#### 4.3.1. Scientific Evidence

After deciding which bolus insulin to start, the next step is to decide what dose to start with, and how to titrate the dose of the bolus insulin in an individualized manner. A number of titration algorithms exist, and one needs to choose an algorithm that best suites the individual. In recent studies, doses of faster aspart and rapid-acting insulin aspart were titrated once-weekly based on pre-meal glucose values, with reduction and addition of 1U, at blood glucose levels of <71 mg/dL and >108 mg/dL, respectively [57,58]. Apart from achieving 2-h PPG within the guideline-defined range, another key consideration of the insulin-dose titration is safety, i.e., avoidance of hypoglycemia. In a study conducted on 876 patients with T2DM with relatively well-controlled diabetes (mean HbA1c 7.55%), glulisine and RHI were administered with NPH, and adjustment of their doses was made as needed to achieve a PPG level of 120–160 mg/dL, while trying to avoid hypoglycemia. The study findings revealed that symptomatic overall, nocturnal, or severe hypoglycemia rates were similar in both groups [92].

Information in Approved Pack Insert on Dose Titration: The approved pack insert of RAIAs and ultrafast-acting insulin (faster aspart) states that the total daily insulin requirement is between 0.5 and 1.0 U/kg/day. In BB treatment regimens, insulin aspart can provide 50–70% of the requirement and the remaining 30–50% should be supplemented by the use of a basal insulin. More frequent glucose monitoring and adjustment of dosage might be necessary if the patients increase the amount of physical activity, change their usual diet, or have a concomitant illness like renal and hepatic impairment [105,107,108,109].

#### 4.3.2. Starting Dose and Titration of Bolus Insulin in Various Guidelines/Recommendations

With respect to dosing and titration of bolus insulin, the starting dose recommended in the ADA guideline is 4 units or 10% of the basal dose, which is adjusted by 1–2 units twice weekly until target blood glucose values are achieved. In the event of a hypoglycemic episode, following the correction of the hypoglycemic state, the underlying cause of the event should be addressed. If there is no clear reason found for the event, the insulin dose may be reduced by 10%−20% [2]. According to the CID expert group guideline, the dose of insulin may be increased by 2 units if the PPG value is between 180 and 220 mg/dL. Furthermore, the dose may be increased by 4 and 6 units if the PPG values are 220–220 mg/dL and >220 mg/dL, respectively [33].

The RSSDI guideline specifies the total daily dose of bolus insulin to be 0.3–0.5 U/kg (40–50% basal: 50–60% bolus). If the 2-h PPG or next pre-meal glucose levels are >180 mg/dL, the bolus dose may be increased by 10% [25].

#### 4.3.3. Expert Group Recommendations 06

Dosing and titration of prandial insulin:

➲ Starting dose: 4 units before the largest meal of the day chosen by the patient. Grade A
➲ Titration based on pre-meal blood glucose (BG) (Criteria A) of the next meal/bedtime or 1- and 2-h PPG (Criteria B)
➲ Frequency of titration: Twice weekly or as deemed relevant by the physician. Grade E

**Titration Algorithm****Criteria A: Pre-meal BG (mg/dL)****Titration**<56−2 *56–84−1 *85–114No adjustment>114+1Criteria B: 1- or 2-h PPG (mg/dL)Titration<1600160–200+2200–220+4>220+6* Dose reduction to be individualized.


**Transfer from Other Insulins**

**Transfer from Other Insulins**

**Dose**
RHI to rapid-acting insulin analogues1:1RHI to fast-acting insulin aspart1:1Rapid-acting insulin analogues to fast-acting insulin aspart1:1Transferring from Premix insulin (30:70)/co-formulation OD to basal-plus regimen70% basal and 30% bolusTransferring from premix insulin/co-formulation BD to basal-bolus regimen40% basal and 20%–20%–20% bolusBD: twice daily; BG: blood glucose; OD: once daily; PPG: postprandial glucose; RHI: regular human insulin. 

## 5. Use of Bolus Insulin in Special Situations and Practical Considerations

### 5.1. Inpatient Hyperglycemia

#### 5.1.1. Scientific Evidence

Hyperglycemia in an inpatient setting could be in a critical or non-critical care setting. There is a wide variability in the results of prevalence of hyperglycemia in critically ill patients. In different studies conducted among patients admitted in critical care, about 30–60% had high blood glucose values. The mortality is higher, and length of hospital stay is longer in patients with hyperglycemia compared to those with normoglycemia [110,111,112,113,114,115]. It is worthwhile to note that, even in the non-critical care setting, hyperglycemia contributes to increased mortality and an increase in length of hospital stay [113,116,117,118]. In a hospital setting, blood glucose values should be maintained in the range of 140–180 mg/dL [2,115,119,120].

In an observational, non-interventional study on hospitalized patients, both patients and physicians (98.6%) favored the use of intravenous (IV) insulin aspart. It was noted that the use of IV insulin aspart reduced blood glucose values from 356.9 mg/dL to 154.9 mg/dL, which is within the ADA 2021 target. The preference for insulin aspart was attributed to the rapid achievement of the target BG, safety, and convenience to shift from IV to subcutaneous (SC) route. No major hypoglycemic events were reported in 99% of the patients [119]. However, this study did not have a comparator arm. Another observational study conducted among 203 Indian patients assessing the safety of IV insulin aspart and RHI in perioperative cardiac setting revealed similar glycemic control (159.87 ± 41.41 mg/dL vs. 160.77 ± 44.39 mg/dL, respectively) and hypoglycemic rates in both groups. However, a better recovery from hypoglycemia with IV aspart was seen when compared to RHI as depicted by lesser time to withhold insulin infusion after a hypoglycemia episode [121]. However, it stands to reason that compared to RHI, the rapid onset of action of insulin aspart is applicable as long as both insulins are being administered subcutaneously; such a difference becomes mute when both are being administered intravenously. As such, one should continue to use RHI for IV administration until such time that a properly designed RCT shows any difference in outcomes or safety between the two insulins.

A randomized trial in an emergency setting was conducted to compare the rush emergency department hyperglycemia intervention (REDHI) protocol versus usual care in ED. REDHI SC insulin aspart protocol with daily detemir showed a significantly lower blood glucose level compared with that provided by usual care (217 mg/dL vs. 257 mg/dL, *p* < 0.001). Additionally, the protocol did not compromise the safety or prolong the length of the hospital stay of patients [122]. A retrospective database analysis of >35,000 nonsurgical patients with 5568 propensity matched pairs comparing subcutaneously administered analogue insulins vs. RHI showed insulin analogues had a modest benefit in achieving glycemic control (relative risk (RR) = 0.88; 95% CI: 0.81–0.95), lower hospital mortality rate (RR = 0.52; 95% CI: 0.45–0.61), and shorter length of stay (LOS) (0.668-day reduction; 95% CI: 0.44–0.89) when compared with human insulin [123]. Among the available insulins, aspart, glulisine, lispro, and faster aspart and RHI are approved for IV use [105,107,108,109,124].

#### 5.1.2. Recommendations for in-Hospital Glycemic Management in Various Guidelines/Recommendations

The ADA guidelines recommend continuous IV insulin infusion in the critical care setting. In the non-critical hospital settings, the guideline suggests use of either RHI or insulin analogues. In situations where oral intake is poor or uncertain, administration of the rapid-acting insulin immediately post-meal servesis a safer alternative. Furthermore, for the management of hyperglycemia, the ADA guidelines recommend the use of either rapid- or short-acting SC insulins before meals or every 4–6 h if the patient is receiving continuous enteral/parenteral nutrition [2]. According to CDA guidelines, for in-patients who are not eating regularly, bolus insulin can be withheld or reduced, and basal insulin continued. The guideline prefers the use of RAIAs as SC bolus and correction insulins [80].

For the critical care setting in the Indian scenario, consensus recommendations suggest the use of intensive IV insulin therapy administering short-acting regular human insulin through an infusion pump. When transiting the route of administration from IV to SC, SC insulin should be administered at least 1–2 h before the discontinuation of IV insulin or 15–30 min if RAIAs are used [115]. In a non-critical setting, for patients who can take oral diet once or twice daily, basal or intermediate-acting (degludec/detemir/glargine U100) insulin and prandial doses of short-acting (regular insulin) or rapid acting (aspart/lispro/glulisine) insulin are the choices of insulin therapy. Non-critical patients on parenteral or enteral nutrition are preferably initiated on basal insulin alone in combination with correctional doses of rapid-acting insulin analogues every 4 h or regular insulin every 6 h [117].

On similar lines, the RSSDI 2017 guideline recommends the preparation for insulin infusion in an intensive care unit with preference of short-acting regular human insulin. When transitioning from insulin infusion to SC insulin and/or OAD, regular human insulin/NPH or short- and long-acting insulin analogues can be used, considering the affordability of the patient. In non-critically ill patients, the guidelines prefer the use of NPH and regular insulin or long-acting and rapid-acting insulin analogues. For renally-compromised patients in non-critical care situation, short-acting insulin analogue is preferred over regular insulin. In cases where patients are on special feeding (enteral feeding), regular insulin at an interval of 6 h is preferred over rapid-acting analogues for their longer duration of action [25].

#### 5.1.3. Expert Group Recommendations 07

➲ Hyperglycemia in hospital setting is associated with significant increase in morbidity, length of hospital stays, and mortality. Grade A
➲ Intravenous administration of RHI with frequent monitoring and titration of insulin dose is necessary to achieve good glycemic control in critically ill patients with hyperglycemia. Grade B
➲ SC RAIAs maybe preferred over SC RHI for management of hyperglycemia in non-critical care hospital setting. Grade A
➲ Subcutaneous fast-acting insulin aspart may be preferred over SC RAIAs for the management of hyperglycemia in hospital setting. Grade E

### 5.2. Hyperglycemia in Pregnancy

#### 5.2.1. Scientific Evidence

It is crucial to select the appropriate type of insulin in pregnant women, to effectively manage glycemia, without exerting any detrimental effect on the growing fetus. Evidence from T1DM pregnant patients has proven insulin aspart to be as safe and effective as human insulin in the BB regimen with NPH insulin. It further offers the benefits of effective PPG control [125]. Similar findings were observed in an RCT with respect to safety in pregnant women with T1DM taking either prandial insulin aspart or RHI; both insulins were safe with no statistically significant difference [126]. In a parallel, open-label study conducted among 322 pregnant women with T1DM, comparison of insulin aspart with RHI in BB therapy with NPH with respect to fetal and perinatal outcomes was performed: insulin aspart regimen reported fewer fetal losses (14 vs. 21) and preterm deliveries (20.3% vs. 30.6%) when compared to RHI [127].

A study analyzed the maternal and cord blood insulin antibody levels in 97 pregnant women with T1DM treated with either insulin aspart or RHI; the findings concluded non-development of insulin antibodies during pregnancy with the use of either of the insulins, and no evidence was found to support placental transfer of insulin aspart [128]. A meta-analysis by Lv et al., which included pregnant women with T1DM, T2DM, and women with gestational diabetes mellitus, did not observe any increase in risk of macrosomia with the use of insulin aspart during pregnancy [129]. The Diabetes in Early Pregnancy Study and other studies conducted among pregnant women showed that elevated 1-h PPG excursions are directly related to macrosomia [130,131]. It is important to note that insulin treated pregnant women based on 1-h PPG value achieved better reduction in HbA1c values and had significantly less macrosomic infants [132,133]. The ADA and ACOG guidelines recommend a target value of 1-h PPG <140 mg/dL for management of hyperglycemia in pregnancy [2,134]. In this context, the early glucose-lowering effect of faster aspart, and significantly better 1-h PPG reduction compared to rapid-acting insulins observed in non-pregnant T1DM and T2DM patients, may have a beneficial role in management of hyperglycemia in pregnancy [58,100,101,102]. Faster aspart is also approved for the management of hyperglycemia in pregnancy [105].

With respect to USFDA classification of insulins for use in pregnancy, regular insulin, insulin aspart, and insulin lispro are categorized as Category B (animal reproduction studies have failed to demonstrate a risk to the fetus and there are no adequate and well-controlled studies in pregnant women) [135]; fast-acting insulin aspart has been approved based on the safety profile of insulin aspart. Furthermore, insulin glulisine has been categorized as Category C (animal reproduction studies have shown an adverse effect on the fetus and there are no adequate and well-controlled studies in humans, but potential benefits may warrant the use of the drug in pregnant women despite potential risks) [135].

#### 5.2.2. Role of Bolus Insulin in the Management of Hyperglycemia in Pregnancy in Various Guidelines/Recommendations

The ADA standards of medical care in diabetes advocates insulin as a safe option for the management of both T1DM and T2DM in pregnancy, as it does not cross the placenta. It further mentions the target levels for gestational diabetes mellitus as: fasting BG < 95 mg/dL and target 1-h PPG < 140 mg/dL or 2-h PPG < 120 mg/dL [2]. The CDA advocates that pregnant women with T1DM or T2DM should receive an individualized insulin regimen and glycemic targets should be adjusted using intensive insulin therapy [136]. Aspart or lispro may be used in women with pre-existing diabetes to improve postprandial blood glucose and reduce the risk of severe maternal hypoglycemia compared with RHI [134].

The DIPSI consensus recommends insulin aspart if PPG is high. Furthermore, it advocates insulin aspart for bolus insulin as it is more effective than human insulin in decreasing PPG concentrations [137].

#### 5.2.3. Expert Group Recommendation 08

➲ Insulin aspart/insulin lispro is preferred over RHI for better PPG control and lower risk of hypoglycemia when managing hyperglycemia in pregnancy with insulin. Grade A
➲ Controlling 1-h postprandial hyperglycemia is especially important in individuals with hyperglycemia in pregnancy to reduce the risk of maternal and fetal complications. Grade A
➲ Fast-acting insulin aspart may be preferred over RAIAs for better control of 1-h and 2-h PPG in hyperglycemia in pregnancy. Grade C

### 5.3. Successful Treatment with Bolus Insulin—Sequential Addition vs. Full-Scale Basal-Bolus Therapy

#### 5.3.1. Scientific Evidence

Starting a Full Scale Basal-Bolus Therapy Compared to Sequential Addition of Bolus Insulin: Full STEP study was conducted to evaluate the outcomes of sequential addition of up to three bolus insulins compared to full scale BB therapy. Stepwise addition of prandial insulin achieved the primary objective of non-inferiority of HbA1c reduction (mean treatment difference −0.14; 95% CI: 0.02–0.30) when compared to full-scale BB therapy. At the end of the study, patients achieving an HbA1c target of <7% were comparable between the two groups (stepwise vs. BB: 55.9% vs. 63.3%). Stepwise prandial insulin dosing compared to full BB dosing, had significantly less overall hypoglycemic events (rate ratio (RR) 0.58 (95% CI: 0.45–0.75); *p* < 0.0001), less dropouts (14% vs. 26%; *p* = 0.002), and better overall patient satisfaction. Additionally, it is recommended to optimize (basal dose) and intensify (addition of bolus doses in steps) treatment when the HbA1c level is ≥7% [138]. Prandial insulins are usually co-administered with a basal insulin and/or OADs. More frequent glucose monitoring with reduction in the dose/modification of OADs may be needed to strike a right balance between effectiveness and safety [139,140].

Right Meal for Bolus Insulin Initiation: A 48-week study compared the stepwise addition of insulin aspart to either the largest meal (where the titration was based on pre-meal glucose values) or to the meal with the largest prandial glucose increment (titration based on post-meal glucose values) in patients with T2DM inadequately controlled on basal insulin and oral OAD. After 12 weeks of basal insulin detemir dosage optimization, participants with an HbA1c level >7% entered three 12-week treatment periods with stepwise addition of a first insulin aspart bolus. This was followed by second and third steps if the HbA1c level remained at >7% after 12 and 24 weeks of treatment, respectively. Both the strategies were found to be equally effective in intensifying therapy. The study reported a decrease ofHbA1cvalue by about 1.2% in both the groups. Postprandial glucose levels were found to be similar in both the groups at 36 weeks of the trial. Furthermore, the number and frequency of hypoglycemic episodes were similar between the two regimens. The weight gain in patients was also similar across both the treatment groups [141].

Timing of Bolus Insulin Administration: In a randomized, double-blind, double dummy, 4-period cross-over study conducted among 20 T1DM patients, the relationship of insulin aspart with meal (immediately before and 15 min after the meal) was compared with RHI (−15 min before and with meal). Insulin aspart administered 15 min after the meal was as effective as RHI administered 15 min before meal in reducing prandial excursions [142]. The ONSET 1 trial was a randomized, multicenter, treat-to-target trial which had three arms comparing mealtime faster aspart (−2 to 0 min with meal) with mealtime insulin aspart (−2 to 0 min with meal) with post-meal faster aspart (20 min after the meal). Mealtime faster aspart showed significantly better HbA1c reduction compared to mealtime insulin aspart (ETD: −0.15% (95% CI: −0.23–−0.07). Post-meal faster aspart was non-inferior to mealtime insulin aspart in terms of HbA1c reduction (ETD: 0.04%; 95% CI: −0.04–0.12) [99,100]. Thus, insulin aspart can be given before or soon after the meal and faster aspart can be dosed just before or up to 20 min after the start of a meal owing to the faster onset of action compared to insulin aspart [101,102,142].

#### 5.3.2. Full-Step vs. Stepwise Basal-Bolus Regimen in Various Guidelines/Recommendations

The AACE 2020 recommends both full-step and stepwise approaches. The guideline recommends starting basal insulin with long-acting insulin and intensifying the regimen by addition of bolus insulin before the largest meal of the day [68]. For successful treatment of patients with T2DM, ADA recommends a sequential stepwise approach to start bolus insulin before the largest meal of the day. In case the HbA1c level is not controlled, the BB regimen may be followed by the addition of ≥2 rapid-acting insulin injections before meals. When initiating combination injectable therapy, metformin therapy should be maintained, while sulfonylureas and DPP-4 inhibitors are typically weaned or discontinued. In patients with suboptimal blood glucose control, especially those requiring large insulin doses, adjunctive use of a thiazolidinedione or an SGLT2 inhibitor may help to improve control and reduce the amount of insulin needed, though potential side effects should be considered [2]. The RSSDI ESI 2020 recommends that it is important to consider reducing the dose of sulphonylureas when prandial insulins are introduced [143]. With regard to timing of the bolus insulin administration, the RSSDI guideline recommends regular insulin to be given about 30 min before meals; RAIAs are suggested to be given just before the meal [25].

#### 5.3.3. Expert Group Recommendations 09

➲ Whenever possible, stepwise addition of bolus insulin is preferred over full basal-bolus therapy in people with T2DM to reduce the risk of hypoglycemia, patient dropouts, and improved patient satisfaction. Grade A
➲ It is recommended to start with administering bolus insulin before the largest perceived meal of the day. Grade A
➲ Post-meal dosing of insulin (up to 20 min), if needed, is an option in individuals: Grade A
  ➢ Who are unable to predict the exact timing or carbohydrate content of a meal in advance (e.g., on social occasions);
  ➢ Experiencing loss of appetite or nausea;
  ➢ Having unpredictable appetite;
  ➢ Who have forgotten to administer an injection pre-meal.
➲ Fast-acting insulin aspart is preferred over RAIAs in individuals who need post-meal insulin dosing. Grade A

There are several strengths in these recommendations. The expert group has thoroughly reviewed the sourced evidence under each section of the consensus framework and the recommendations of a few major local and international diabetes and endocrinology associations. Various aspects of use of bolus insulin in people with T2DM were then discussed at length and, together with the collective clinical experience of the members of the expert group, these easy to follow recommendations for the effective use of bolus insulin in the management of people with T2D requiring insulin were drafted. These recommendations provide a comprehensive overview of the topic, identify gaps in research, and bridge them with clinical experience-based recommendations. A major limitation of these recommendations is the lack of a systematic review of the literature. Although all attempts were made to identify all relevant literature on this topic, it is possible that a few studies have remained unidentified.

## 6. Conclusions

The contribution of PPG excursions to the increased risk of micro- and macrovascular complications in individuals with T2DM is well established. As such, attention should be paid to controlling both FPG and PPG in the management of T2DM. A number of OHAs target PPG. However, when OHAs fail to achieve PPG targets in individuals with T2DM, bolus insulin is necessary to achieve PPG control. Over the years, there has been a continued innovation in bolus insulins, starting from short-acting human insulins (RHIs) to RAIAs (lispro/aspart/glulisine) to ultrafast-acting insulins (faster aspart). These innovations have been oriented towards developing a bolus insulin that better mimics physiological mealtime insulin secretion, allowing more mealtime flexibility and improved safety in terms of reduction of hypoglycemic events thereby enabling better achievement of PPG and HbA1c goals.

This consensus statement is intended to serve as a reference for physicians and specialists for the effective use of bolus insulin in the management of individuals with T2DM. It is hoped that this document shall lead to a reduction in the inertia among physicians in initiating BB therapy, allowing better glycemic control in insulin-requiring people with T2DM.

We also hope that these consensus recommendations not only serve as a useful resource for the physicians in the day-to-day care of people with T2DM, but also serve as a trigger for further research on the use of bolus insulins in the management of T2DM.

## Figures and Tables

**Table 1:** Characteristics of prandial/bolus insulin preparations [27,28].

Insulin Preparations	Onset of Action (min)	Peak Action (h)	Duration of Action (h)
Short-acting			
Regular insulin	30–60	1–3	6–10
Rapid-acting			
Insulin lispro	15–30	0.5–1	3–5
Insulin aspart	10–20	0.5–1	4–5.3
Insulin glulisine	15–14	0.5–1	4–6.3
Ultrafast-acting			
Fast-acting insulin aspart	4.9 min earlier *	10.5 min earlier *	14.3 min shorter *

* comparison with insulin aspart.

**Table 2:** Evidence-grading system [2,34,35].

Level of Evidence	Description
A	Clear evidence from well-conducted, generalizable, randomized controlled trials that are adequately powered, including: evidence from a well-conducted multicenter trial; evidence from a meta-analysis that incorporated quality ratings in the analysis. Supportive evidence from well-conducted, randomized, controlled trials that are adequately powered, including: evidence from a well-conducted trial at one or more institutions; evidence from a meta-analysis that incorporated quality ratings in the analysis. Strong recommendations from key national or international guidelines, including RSSDI, ADA, IDF, and AACE guidelines, supported by level A evidence.
B	Supportive evidence from well-conducted cohort studies: evidence from a well-conducted prospective cohort study or registry; evidence from a well-conducted meta-analysis of cohort studies. Supportive evidence from a well-conducted case-control study.
C	Supportive evidence from poorly controlled or uncontrolled studies: evidence from randomized clinical trials with one or more major or three or more minor methodological flaws that could invalidate the results; evidence from observational studies with high potential for bias (such as case series with comparison with historical controls); evidence from case series or case reports. Conflicting evidence with the weight of evidence supporting the recommendation.
E	Expert consensus or clinical experience.

AACE: American Association of Clinical Endocrinologists; ADA: American Diabetes Association; IDF: International Diabetes Federation; RSSDI: Research Society for the Study of Diabetes in India.

**Table 3:** Recommendations for time to check and target for PPG in various guidelines [2,16,24,25,68,75,76,77,78,79].

Guidelines	Recommendations (Time after Meal)	Recommendations (PPG Target)
ADA 2021	1–2 h	<180 mg/dL
IDF 2014	1–2 h	<160 mg/dL
CDA 2018	2 h	90–180 mg/dL; If HbA1c target is not achieved, target may be reduced to 90–144 mg/dL—Balanced against risk of hypoglycemia
RSSDI 2017	1–2 h	<160 mg/dL
AACE 2020	2 h	<140 mg/dL
ICMR 2018	2 h	120–140 mg/dL
Australia 2009	2 h	106–180 mg/dL
Sri Lanka	2 h	80–145 mg/dL
Malaysia	2 h	<140 mg/dL

AACE: American Association of Clinical Endocrinologists; ADA: American Diabetes Association; CDA: Canadian Diabetes Association; HbA1c: glycosylated hemoglobin; ICMR: Indian Council of Medical Research; IDF: International Diabetes Federation; PPG: Postprandial glucose; RSSDI: Research Society for the Study of Diabetes in India.

**Table 4:** Options for intensification after basal insulin therapy.

Options	Efficacy	Hypos	Weight Change	Cost
Basal plus insulin regimen	Highest—with greater flexibility but increased complexity	+++	++	Depends on the insulin used
Premixed insulin regimen	Highest—low complexity but lesser flexibility	++	+	Depends on the insulin used
Adding GLP-1RA	High—with highest flexibility and least complexity	+	Reduction	High compared to other two regimens

CV: Cardiovascular; GLP-1RA: Glucagon-like peptide-1 receptor agonist.

## Data Availability

Not applicable.
